# Evaluation of Policy Effectiveness by Mathematical Modeling for the Opioid Crisis with Spatial Study and Trend Analysis

**DOI:** 10.3390/healthcare9050585

**Published:** 2021-05-14

**Authors:** Jiaji Pan, Shen Ren, Xiuxiang Huang, Ke Peng, Zhongxiang Chen

**Affiliations:** 1 College of Engineering and Design, Hunan Normal University, Changsha 410081, China; Pan.jiaji@hunnu.edu.cn (J.P.); hxxpxj01@163.com (X.H.); pengke@hunnu.edu.cn (K.P.); 2 Center for Cryo-Biomedical Engineering and Artificial Organs, Department of Mechanical Engineering, University of Washington, Seattle, WA 98195, USA; shenren8@uw.edu

**Keywords:** opioid crisis, entropy weight method, rank sum ratio method, autoregression model, policy evaluation

## Abstract

The current opioid epidemic in the US presents a great problem which calls for policy supervision and regulation. In this work, the opioid cases of five states were used for trend analysis and modeling for the estimation of potential policy effects. An evaluation model was established to analyze the severity of the opioid abuse based on the entropy weight method (EWM) and rank sum ratio (*RSR*). Four indexes were defined to estimate the spatial distribution of development and spread of the opioid crisis. Thirteen counties with the most severe opioid abuse in five states were determined using the EWM-RSR model and those indexes. Additionally, a forecast of the development of opioid abuse was given based on an autoregressive (AR) model. The *RSR* values of the thirteen counties would increase to the range between 0.951 and 1.226. Furthermore, the least absolute shrinkage and selection operator (LASSO) method was adopted. The previous indexes were modified, incorporating the comprehensive socioeconomic effects. The optimal penalty term was found to facilitate the stability and reliability of the model. By using the comprehensive model, it was found that three factors—VC112, VC114, VC115—related to disabled people have a great influence on the development of opioid abuse. The simulated policies were performed in the model to decrease the values of the indicators by 10%–50%. The corresponding *RSR* values can decline to the range between 0.564 and 0.606. Adopting policies that benefit the disabled population should inhibit the trend of opioid abuse.

## 1. Introduction

The widespread nature of cases of the misuse of and addiction to opioids, including prescription pain relievers, heroin, and synthetic opioid cases such as Fentanyl, is a serious national crisis that affects public health as well as social and economic welfare. The Center for Disease Control and Prevention estimates that prescription opioid cases misuse alone brings a loss of approximately USD 78.5 billion a year to the United States [1]. The rapidly emerging crisis, which has resulted in a significant loss of lives, calls for a coordinated, comprehensive and multidisciplinary response. In response to the opioid cases crisis, many measures have been taken by the U.S. Department of Health and Human Services to mitigate opioid abuse across the country. However, rates of opioid overdose remain severe. A total of 46,802 opioid overdose deaths occurred in 2018, accounting for 69.5% of all drug overdose deaths [2].

It has been noticed that opioid overdoses are regionally related. Many researchers analyzed the spatial difference in opioid overdoses, illustrating the variation in different states [3] as well as between rural and urban areas [4]. Analysis with geographic tools was conducted to study the environmentally correlated factors of opioid overdose and the related deaths, such as the percentage of disabled people or the population percentage of a specific race [5]. A difference in the spatial pattern of the opioid epidemic should be demonstrated as a result of considering socioeconomic factors, such as income [6] and population density [7]. The geographical clustering and spread of opioid cases have been the focus of studies. Some research using SatScan [8], a software used to deal with spatial–temporal data, was carried out to generate clusters adopting models such as those of Poison or Bernoulli [9,10,11]. The clusters formulated by spatial–temporal data were used to analyze the factors correlated with opioid case outcomes.

By elucidating the effects of associated issues on opioid spread, relevant policies could be adjusted necessarily to prevent the worsening of opioid abuse. Certain trend analyses have implied the corresponding policy changes [12]. The enforced policy and administration should play an effective role in preventing the development of opioid abuse. The mortality rate due to opioid overdose has been successfully reduced by police actions [13]. Hierarchical Bayesian Poisson space–time models were used to investigate the interactions between socioeconomic conditions and prescription opioid overdose or heroin overdose situations [14]. Often, space–time statistical models could be used to understand the effect of local policies [15,16]. Stopka et al. proposed logic models to relate the policies to the spatial distribution of the opioid crisis [17]. The effect of policy was predicted to have been targeted at specific groups.

Davis and Carr analyzed the law related to Naloxone access and suggested provisional changes in some states to reduce overdose-related morbidity or mortality [18]; though some models revealed that these laws should have less state-level effects [19]. Such an analysis could be used to guide the revised interventions to curb the trend of the opioid epidemic. A further understanding of the regional differences can help in policy responses [20], as could trend analysis in the abuse and misuse of opioids [21].

Additionally, opioid abuse and misuse could be predicted using appropriate models [22]. By analyzing opioid abuse trends, and combining the correlating spatial factors, opioid abuse should be manageable and be brought under control through relevant policy changes. Other than the unequal spatial factors, the emerging opioid crisis was affected by economic disparities [23], which should be considered in the intended intervention polices. Although the trend analysis and modeling could reveal considerable information to guide the regulation and authority efforts, less attention has been given to developing a model to estimate the possible effects of the proposed policy. The evaluation of policy could be an effective approach to solve the complex and dynamic opioid epidemic problem [24]. Policy makers should consider strategies under the assistance of computational analysis and predictions [25].

In this study, we evaluated areas where opioids are rampant in five states of United States. The indicators associated with the severity of opioid abuse were defined to build a comprehensive evaluation model. The model incorporated the historical data and demographic characteristics of the regions to analyze the opioid abuse areas. The particular indexes for counties in several states were obtained with regard to the degree of the opioid abuse and spread. Further promoted by time series methods, the model was used to estimate the possible effects of the proposed policies targeted at the vulnerable population. The associated index change in the model could be used to evaluate the policy effectiveness, which can assist governmental departments in adjusting policies to suppress the spread of opioids.

## 2. Methods

### 2.1. Data Source and Preprocessing

The data included drug reports provided by the Drug Enforcement Administration (DEA) (Table S1) and U.S. Census population data from 2010 to 2016 (Tables S2–S8). The descriptions of the variables in the U.S. Census population data table (Tables S2–S8) are listed in Table S9. Drug identification counts during the period 2010–2017 for narcotic analgesics (synthetic opioid cases) and heroin in each of the counties from these five states, Virginia (VA), Pennsylvania (PA), Kentucky (KY), Ohio (OH), West Virginia (WV) were collected by the National Forensic Laboratory Information System (NFLIS) of the DEA (Table S1). The drug reports (DR), total county drug reports (TDRC) and total state drug reports were used for analysis. The statistical standards changed in the year 2013. To perform a coherent analysis, the EWM-RSR model described in the following section was established based on the data from 2013 to 2016. When data were not available for some variables, average values of the variables were filled in the missing place for the modeling process.

### 2.2. EWM-RSR Evaluation Model

Several indicators were defined to assess the level of opioid abuse in this section. We then conducted a comprehensive evaluation using an entropy weight method (EWM) and a rank-sum ratio (*RSR*) method.

The entropy method based on information theory was an objective weighing method. The weight of indexes defined in the method were determined based on the degree of order of the information contained in each index. The method evaluated the dispersion degree of the data, which was the most commonly used objective method of weighing. Entropy was a measure of uncertainty of the indexes. A greater uncertainty corresponds to a smaller entropy value. Thus, the entropy weight of the indicator was larger if it contained more information.

To evaluate the severity of the development of opioids in county *i*, we selected $\rho_{i}$, $q_{i}$, $Q_{i}$, and $LOFC_{k}$ as the evaluation indicators. $\rho_{i}$ represented the proportion of opioid cases in all drug use cases for a county *i*. $q_{i}$ represented the average change rate in opioid cases for a county *i*. $Q_{i}$ represented the average change rate in the proportion of opioid cases use. $LOFC_{k}\left(i\right)$ represented the odds ratio of the county *i* opioid cases and the sum of opioid cases in its surrounding *k* distance neighboring counties. These indicators demonstrated positive effects on the severity of the development of opioids. The higher value of the indicators corresponded to a more serious development of opioids in the county *i*.

The rank-sum ratio (*RSR*) method [26] was a statistical analysis method applied in the multi-index comprehensive evaluation of statistical prediction, classification, statistical quality control and many scientific aspects. It was a non-parametric evaluation method which considered the relative magnitude of the indicators to be evaluated. In this study, the socioeconomic development of each county was imbalanced with unique characteristics. Thus, the *RSR* method was adopted since it is difficult to directly determine the relationship between the magnitude of each indicators.

#### 2.2.1. Spread Index of the Model

**Definition** **1.** *The proportion of opioid cases in all drug use case $\rho_{i}^{t}$.*

Based on the data used for analysis, the percentage of opioid cases is defined in county *i* in the year *t* by

(1) $$\rho_{i}^{t}=\frac{\sum_{m}DR_{i,m}^{t}}{TDRC_{i}^{t}},$$

where *m* represents one kind of opioid such as morphine, codeine or heroin. DR represents the drug report related to opioid case. TDRC represents the total drug reports of a county. The average ratio of the county *i* in 2010 to 2017 is defined as

(2) $$\rho_{i}=\frac{1}{8}\sum_{t=2010}^{2017}\rho_{i}^{t}.$$

**Definition** **2.** *The average rate of change of opioids use for the county i $q_{i}$ is:*

(3) $$q_{i}=\frac{\frac{1}{4}\sum_{t=2014}^{2017}\sum_{m}DR_{i,m}^{t}-\frac{1}{4}\sum_{t=2010}^{2013}\sum_{m}DR_{i,m}^{t}}{\frac{1}{4}\sum_{t=2014}^{2017}t-\frac{1}{4}\sum_{t=2010}^{2013}t}$$

The rate of change in opioid cases may directly reflect the growth rate of opioid cases in a county. Calculation with the subtraction of data from adjacent years should lead to a large error. Hence, the data of the total use of opium cases from 2010 to 2013 were compared with those of the total use of opioid cases from 2014 to 2017.

**Definition** **3.** *The average rate of change in the proportion of opioid cases $Q_{i}$ is:*

(4) $$Q_{i}=\frac{\frac{1}{4}\sum_{t=2014}^{2017}\rho_{i}^{t}-\frac{1}{4}\sum_{t=2010}^{2013}\rho_{i}^{t}}{\frac{1}{4}\sum_{t=2014}^{2017}t-\frac{1}{4}\sum_{t=2010}^{2013}t}$$

The average rate of change in the proportion of opioid cases could indicate the proportion of drug users who prefer opioids. To measure the severity of opioid cases in a county, both time and space should be taken into consideration. We defined two indicators. One was the change of opioid cases of the counties between 2010 and 2017. The other one was the ratio of the number of opioid cases of a county to that of its surrounding counties. By these two indicators, we could measure the level of opioid use cases in a county. Therefore, the distance between the county *i* and the county *j* was defined to determine the indicators. In order to show the using level of opioid cases in the county relative to the neighboring county, the local outlier factor (LOF) [15,27] was adopted to define the county *i* relative to the neighbor county’s odds ratio.

**Definition** **4.** *The distance between the county i and the county j d(i,j) is:*

(5) $$d\left(i,j\right)=\frac{\pi R}{180}\cdot \arccos\left(\sin\varphi_{i}\sin\varphi_{j}\cos\psi_{i-j}-\cos\varphi_{i}\cos\varphi_{j}\right)$$

The position of each county was determined using the latitude and longitude coordinates found in Google Maps. According to the spherical distance formula, the distance between the county *i* and the county *j* could be obtained. $\varphi_{i}$ and $\varphi_{j}$ represented the remnants of the latitude of the county *i* and the county *j*, respectively. $\psi_{i-j}$ represented the longitude difference between the county *i* and the county *j*.

**Definition** **5.** *$d_{k}\left(i\right)$ represented the distance between the county i and the k-th nearest county to the county i.*

**Definition** **6.** *U(i,k) was the k Neighborhood County Set of the county i if there exists a distance order $d\left(i,j_{1}\right)\leq d\left(i,j_{2}\right)\leq \cdots \leq d\left(i,j_{k}\right)=d_{k}\left(i\right)$, where $j_{l}\in U\left(i,k\right),l=1,\ldots ,k\;\mathrm{and}\;j_{l}\neq i$. There were k counties in U(i,k) which satisfied the distance between the county in U(i,k) and the county i less than or equal to $d_{k}\left(i\right)$.*

**Definition** **7.** *The odds ratio of the county i opioid cases and the sum of opioid cases in its surrounding k distance neighboring counties: $LOFC_{k}\left(i\right):$*

(6) $$LOFC_{k}\left(i\right)=\frac{\sum_{t=2010}^{2017}\sum DR_{i,m}^{t}}{\sum_{j\in N_{k}\left(i\right)}\sum_{t=2010}^{2017}\sum_{m}DR_{j,m}^{t}}$$

Due to differences in economic and medical standards in each county, we did not evaluate the severity of opioid use merely based on the number of opioid cases within a single county. Considering the circulation of opioids, we defined the ratio of the number of opioid cases in a county to that of its surrounding neighborhoods to evaluate the relative use of opioids in that county.

#### 2.2.2. Calculation Steps

The calculation steps of the model were as follows:

(1) Rank the original dataset

The *m* evaluation indicators of the *n* evaluation objects were arranged into an original data table with *n* rows and *m* columns, followed by ranking each indicator corresponding to each object. The benefit indicators were ranked from small to large. The cost indicators were ranked in reverse order. Data for the same indicator were averaged. Thus, the rank matrix was achieved and denoted as $R={\left(R_{ij}\right)}_{m\times n}$. Subscript *i* was the indicator and *j* represented the object to be evaluated.

(2) Determine indicator weights

The main calculation steps were as follows: A total of *n* objects were to be evaluated. Each object had *m* indicators, which resulted in a n×m matrix. $x_{ij}$ was the value of the *i*-th indicator corresponding to the *j*-th object. To perform the analysis, the modified value in the matrix ${\tilde{x}}_{ij}$ was set to be positive by subtracting the minimum value of each indicator from $x_{ij}$:

(7) $${\tilde{x}}_{ij}=x_{ij}-\min\left\{x_{1j},x_{2j},\cdots x_{nj}\right\}$$

Then, the weight of the indicators was normalized:

(8) $$p_{ij}=\frac{{\tilde{x}}_{ij}}{\sum_{i=1}^{n}{\tilde{x}}_{ij}}$$

The entropy of each indicator was achieved after normalization:

(9) $$e_{j}=-\frac{1}{\ln n}\sum_{i=1}^{n}p_{ij}\ln p_{ij},\left(j=1,2,\cdots ,m\right)$$

where $\lim_{p_{ij}\to 0}p_{ij}\ln p_{ij}=0$. Thus, the entropy weight coefficient *h* of each indicator was achieved:

(10) $$h_{j}=\frac{1-e_{j}}{\sum_{j=1}^{m}\left(1-e_{j}\right)},\left(j=1,2,\cdots ,m\right)$$

The larger the entropy weight coefficient $h_{j}$, the more information this indicator carries, illustrating a greater role in the processing of comprehensive evaluation.

(3) Calculate the value of *RSR* and determine the spread

In the *RSR* method, the rank sum was calculated without considering the specific value:

(11) $$RSR_{i}=\frac{1}{n}\sum_{j=1}^{m}h_{j}R_{ij}$$

where $h_{j}$ was the weight of the *j* evaluation indicator and $\sum_{j=1}^{m}h_{j}=1$. The frequency distribution of the *RSR* follows a normal distribution. It can be converted using the Probit model (a generalized linear model). The conversion method was:

**Step** **1.** The *RSR* frequency distribution table was prepared. The frequency of each group *f* was listed. The cumulative frequency ∑f of each group was calculated;
**Step** **2.** The range of ranking and average value of *RSR*s of each group were determined;
**Step** **3.** The cumulative frequency $\bar{R}/n\times 100\%$ was determined. The last item of the cumulative frequency was corrected, denoted as $1-\frac{1}{4n}$;
**Step** **4.** The cumulative frequency to Probit was converted, where Probit was the standard normal distribution *u* corresponding to the cumulative frequency plus five.

(4) Perform subsection insertion sorting for corrected *RSR* value

A linear regression equation was constructed. The cumulative frequency Probit was the independent variable. The modified *RSR* was the dependent variable:

(12) RSR=a+b×Probit

The regression equation was used to calculate the estimated RSR value. Then, the evaluation object was sorted.

### 2.3. Autoregressive Model

The opioid use of each county was assessed to forecast the future development of opioid abuse. The autoregressive (AR) model [28] was used in time series analysis for prediction. The AR model was commonly used to fit stationary sequences model with a time characteristic. Our model considered that the value of $x_{t}$ was primarily affected by the previous *p* period. *x* was one of the indicators to be predicted. Subscript *t* represents a specific period. The following form of AR equation was established by the correlation between the previously collected data and the future expected value:

(13) $$x_{t}=\varphi_{1}x_{t-1}+\varphi_{2}x_{t-2}+\cdots +\varphi_{p}x_{t-p}+\varepsilon$$

where $\varphi_{i},\;i=1,\;\cdots ,\;p$ are the parameters of the AR model that need to be determined. ε represents the error between the predicted value and the real value. This model was named the *p* order AR moving average model, abbreviated as AR(p).

### 2.4. Important Variables Search with LASSO Regression

The introduced indicators were based on historical data to assess the level of opioid abuse from different perspectives. The severity of opioid abuse should be the result of the comprehensive effect of policies, economy and many other factors in the county. Thus, the next step of this model was to identify the factors that may have an influence on opioid abuse by evaluating external indicators.

#### 2.4.1. Normalization of the Indicators

The indicators in the U.S. Census socioeconomic data (Tables S2–S8) were “Estimate”, “Margin of Error”, “Percent” and “Percent Margin of Error”. We selected “Percent” as the value of the indicators. To unify dimensions and facilitate algorithm convergence, all data were normalized before fitting:

(14) $$x_{i}^{*}={\left(x_{i1}^{*},x_{i2}^{*},\cdots ,x_{im}^{*}\right)}^{T}$$

(15) $$x_{ij}^{*}=\frac{x_{ij}-\min\left\{x_{1j},x_{2j},\cdots ,x_{rj}\right\}}{\max\left\{x_{1j},x_{2j},\cdots ,x_{nj}\right\}-\min\left\{x_{1j},x_{2j},\cdots ,x_{rj}\right\}}$$

(16) $$y_{i}^{*}=\frac{y_{i}-\min\left\{y_{1},y_{2},\cdots ,y_{n}\right\}}{\max\left\{y_{1},y_{2},\cdots ,y_{n}\right\}-\min\left\{y_{1},y_{2},\cdots ,y_{n}\right\}}$$

#### 2.4.2. LASSO Model

LASSO [29], also known as the least absolute shrinkage and selection operator, was a method used for variable selection and shrinkage in the medium-or high-dimensional environment. Post-LASSO was to apply ordinary least squares to the model selected by the first-step LASSO procedure. A penalty term was also included in the objective function, instead of adopting a cost function that merely focused on the square error between the prediction and the actual value. In this work, a regression function was established:

(17) $$\hat{y}=x^{*T}\beta$$

where β was a parameter vector in the LASSO model associated with the indicator variables. It was expected to obtain:

(18) $$\min_{\beta }\frac{1}{n}\sum_{i}^{n}{\left(y_{i}^{*}-x_{i}^{*T}\beta \right)}^{2}+\lambda {∥\beta ∥}_{1}$$

where λ was the penalty term. The value of λ determined the number of final variables reserved. When solving the problem, the leave-one-out-cross-validation (LOOCV) method was used to determine the optimal λ.

## 3. Results and Discussion

### 3.1. Trend Analyses of the Amount of Drugs of Five States

**Figure 1:**
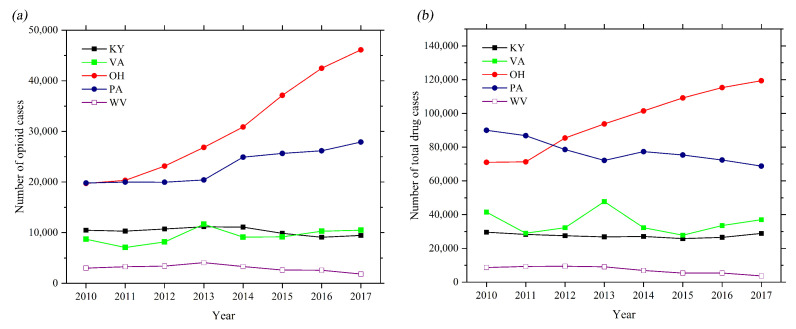
(**a**) Trends of opioid cases of five states; and (**b**) trends of the total drug cases of five states.

As can be seen in Figure 1, the number of opioid cases of OH is highest among the five states, followed by that of KY. The trend of number of opioid cases of VA and PA are similar. The number of opioid cases of WV is lowest. From the trend of drug identification count versus time, the total amount of opioid cases, as well as all drug cases, in OH steadily increases from 2010 to 2017. In contrast, the total drug identification count of PA slowly declines from 2010 to 2013, followed by a slightly increase in 2014, however, it starts to decrease again afterwards. The trend of the total amount of opioid cases and all drug cases of VA fluctuate to a certain degree with a similar variation pattern. There is no tremendous change in terms of opioids and total drug cases in WV and KY.

### 3.2. Heat Maps of the Opioid Cases

**Figure 2:**
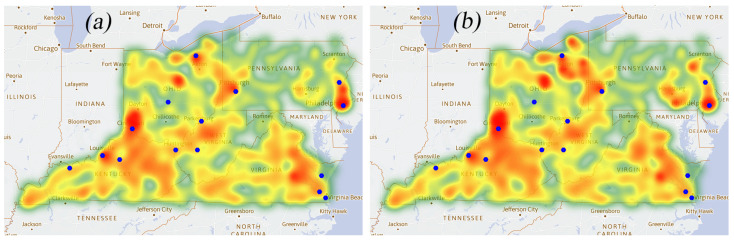
(**a**) Heat map of opioid cases during the period 2010–2013; and (**b**) heat map of opioid cases during the period 2014–2017.

**Figure 3:**
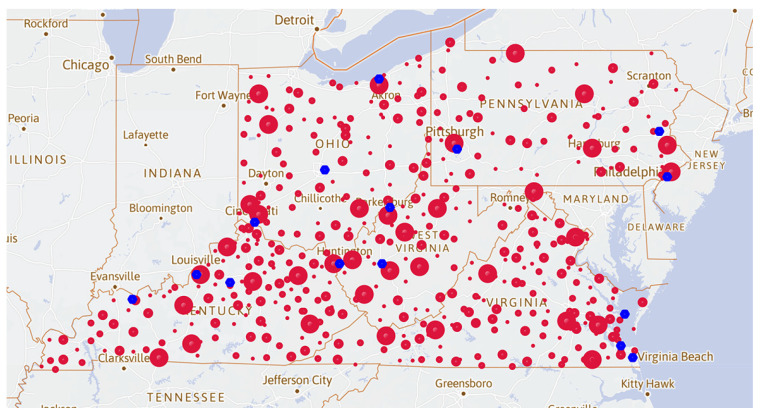
Advantage point distribution map. The size of the point represents the value of LOFC for each county.

**Figure 4:**
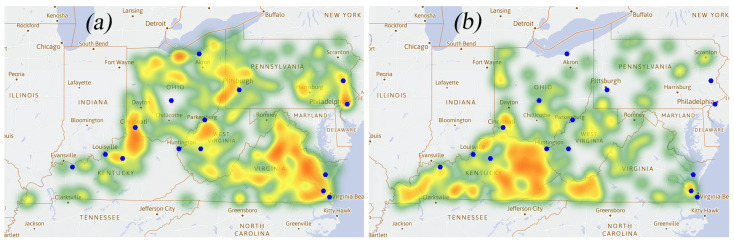
Heat map of $Q_{i}$: (**a**) the distribution of positive $Q_{i}$; (**b**) the distribution of negative $Q_{i}$. Positive $Q_{i}$ illustrate may reflect the effectiveness of regulation.

Based on the radiation direction of the heat map (Figure 2), the occurrence of opioids demonstrates a tendency to spread from the dense areas to the surrounding areas. The three largest cities in each state by population are denoted as blue dots in Figure 2 (these cities are also represented by blue dots in Figure 3 and Figure 4). The three largest cities in five states by population include Columbus, Cleveland, Cincinnati (OH); Virginia Beach, Norfolk, Chesapeake (VA); Louisville, Lexington, Owensboro (KY); Philadelphia, Pittsburgh, Allentown (PA); Charleston, Huntington, Parkersburg (WV). According to the energy distribution of the heat map, the opioid cases are concentrated in the surrounding areas near the large cities. The overall growth rate of opioid cases decreased during the period 2014–2017. It may be assumed that the spread of opioids has been controlled to a certain extent, which could be further validated by the distribution of $Q_{i}$. There has not been a significant increase under these circumstances. However, the areas in northeast KY and southwest OH show relatively significant growth. The development of opioids in some counties has not been suppressed.

### 3.3. Advantage Point Distribution Map of the Opioid Cases

Using the odds ratio defined, an LOFC map (Figure 3) is plotted, where the size of the red dots represents the magnitude of the LOFC values for each county. At the same time, we divided the LOFC size into four levels. It can be seen that absolute advantage points and advantage points are relatively few, compared to the common points and minor points. The total advantage points may represent the counties with relatively sufficient opioids resources and weak government supervision. Secondly, most cities previously denoted in Figure 2 are usually associated with larger and denser absolute advantage points. The total advantage points may represent either more abundant opioid resources or weaker government supervision. Due to multiple factors such as demographic characteristics and governmental regulation, opioid cases are more likely to occur in some counties compared to other surrounding counties.

### 3.4. Heat Map of $Q_{i}$

$Q_{i}$ represents the average change rate in the proportion of opioid cases use, which illustrates the development of opioid cases and the effectiveness of the policy supervision to some certain degree. The positive $Q_{i}$ may suggest a loose or less effective control. As can be seen in Figure 4a, the positive $Q_{i}$ areas almost collide with the area where opioid cases are concentrated (Figure 2), suggesting more effective control may be needed in the major large cities, though the rural area may lack adequate harm reduction services [17]. On the contrary, the negative $Q_{i}$ distributed areas (see Figure 4b) are generally not close to the cities.

### 3.5. Application of the EWM-RSR Model

**Figure 5:**
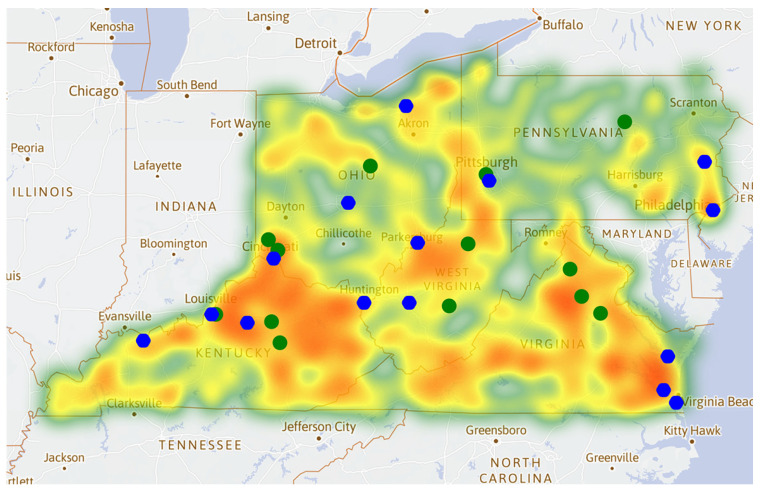
The 13 counties with the most serious opioid abuse.

The EWM-RSR model was used to analyze the severity of the opioid abuse, by considering the different political, economic, and demographic factors affecting the regional development of these five states. The degree of opioid abuse in the given counties was divided into six levels according to the distribution of RSR. The first level indicates that the opioid abuse in the county is very serious. The 13 most serious opioid abuse counties derived from the EWM-RSR model are listed in Table 1. These counties include: Adams, Montgomery, Butler (OH); Allegheny, Lycoming (PA); Madison, Fayette, Jefferson (KY); Warren, Spotsylvania, Culpeper (VA); Nicholas, Harrison (WV), which are denoted with green dots (Figure 5). The blue dots represent the three largest cities in each state.

The $\rho_{i}$, $q_{i}$, $Q_{i}$ and $LOFC_{k}\left(i\right)$ values in the 13 counties with severe opioid abuse are relatively large, indicating the higher proportion of opioid cases and faster growth rate. Drug users in these counties may prefer opioids. In the next step, these counties are used for further analysis.

### 3.6. Application of the AR Model

The time series models were used to make predictions for each of the counties in the five states, and the following two aspects of prediction information were obtained: First, we obtained the predicted value of the total number of crimes in the county by the AR(p) model; Then, the predicted value of the number of opioid cases in the county was obtained by the AR(p) model.

Based on the results of the time series model and the EWM-RSR model, the level of opioid cases abuse could be estimated in each county. Since our time series model defines a period of 4 years, we can speculate on the trend for the next four years. Before 2021, the following counties may enter the stage of cases of the serious abuse of opioids. The specific results are shown in Table 2.

### 3.7. Application of the Comprehensive Evaluation Model

**Figure 6:**
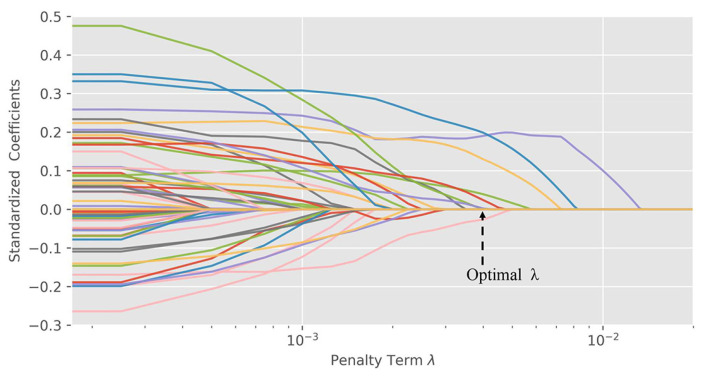
Regularized ridge coefficients.

Figure 6 shows the ridge trace of the LASSO regression of the indicators and variables related to demographic characteristics. The lines in Figure 6 (ridge traces of the LASSO model) represent the variation of the coefficients of the variables(demographic factors) with the increase of penalty term λ. The purple lines correspond to the factor HC03_VC112; the blue lines correspond to the factor HC03_VC197; the red lines correspond to the factor HC03_VC48,the pink lines correspond to the factor HC03_VC135; the orange lines correspond to the factor HC03_VC115; the green lines correspond to the factor HC03_VC162; the black lines correspond to the factor HC03_VC17. By using the LOOCV method, the optimal λ value $3\times 10^{-3}$ was achieved. The reliability and stability of the model were improved by using this lambda value, as well as the regression coefficients in the model.

**Figure 7:**
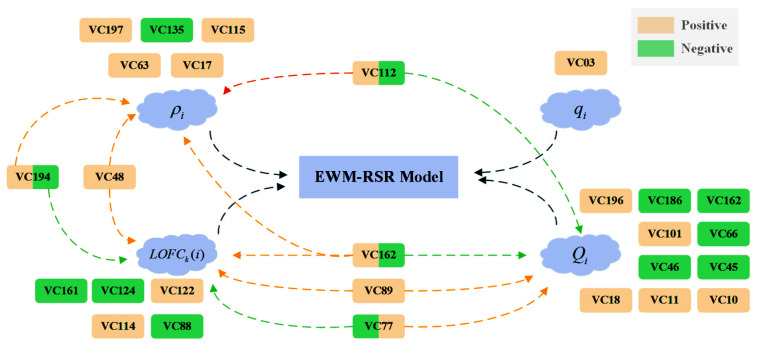
Factor correlation structure diagram.

By means of the LASSO regression, variables were obtained in terms of the demographic characteristics related to the four indicators. The correlation was presented between the variables with large correlation coefficients and four indicators through the factor correlation structure diagram as shown in Figure 7.

To further explore the relationship between the variables describing demographic characteristics and the four indicators, we selected the variables with higher correlation coefficients. The causes of the correlations were analyzed. The analysis results are shown in Table 3 which contains the main part of correlations and explanations. Table S10 includes all the correlations and explanations for the reasons for the remaining variables with small correlation coefficients.

### 3.8. Model Modification with the LASSO Regression Method

A regression equation is established between the population indicators and the evaluation indicators via LASSO regression. Combined with the regression equation established, the model is modified into comprehensive indicators that involve both demographic and historical characteristics:

(19) $$\left\{\begin{array}{c} {\tilde{\rho }}_{i}={\hat{\rho }}_{i}^{*}\cdot \rho_{i}\\ {\tilde{q}}_{i}={\hat{q}}_{i}^{*}\cdot q_{i}\\ {\tilde{Q}}_{i}={\hat{Q}}^{*}\cdot Q_{i}\\ {\tilde{LOFC}}_{k}\left(i\right)={\hat{LOFC}}_{k}{\left(i\right)}^{*}\cdot LOFC_{k}\left(i\right) \end{array}\right.$$

Assuming that the population indicators of all counties remain the same, then drug abuse in the counties depends on their performance in recent years. If the indicators are different, the correction value, such as ${\hat{\rho }}_{i}^{*}$, could be interpreted as the difference of trend and process in the development of drug abuse. After applying the indicator modification in the EWM-RSR model, the impact of opioids can be evaluated more comprehensively on regional historical development and demographic characteristics. According to the deductions listed in Table 3, it was found that there were three factors directly related to disabled people, namely HC03_VC112, HC03_VC115 and HC03_VC114. In the meantime, these indicators played a positive role in increasing the levels of opioid abuse. In response to this situation, policies may be necessitated to increase the care of disabled people and rationally control the use of their opioids.

According to the model, the change of demographic characteristics in a short period of time would have a weak impact on historical development but ultimately would affect its development speed and trend. Let this effect be generated at a rate of α. The original demographic feature is ${\hat{\rho }}_{i,\;\mathrm{old}\;}^{*}$. The new demographic characteristics under natural influence or policy impact are ${\hat{\rho }}_{i,\;\mathrm{new}\;}^{*}$. Then, the effect equation is defined for comprehensive evaluation under the new demographic characteristics:

(20) $${\hat{\rho }}_{i,\;\mathrm{new}\;}=\left[\alpha \left({\hat{\rho }}_{i,\;\mathrm{new}\;}^{*}-{\hat{\rho }}_{i,\;\mathrm{old}\;}^{*}\right)+{\hat{\rho }}_{i,\;\mathrm{old}\;}^{*}\right]{\hat{\rho }}_{i,\;\mathrm{old}\;}$$

The effectiveness of the policy could be evaluated by whether the value of EWM-RSR is reduced using the modified comprehensive model. This change in the evaluation should be superior to the original time series model that relies on the historical data.

### 3.9. Simulation and Estimation of Policy Effectiveness

To determine the effectiveness of the simulated policies, the four indicators established by all the counties are averaged as the indicators of five states. Assuming that the simulated policies have been implemented, the variables VC112, VC115, VC114 related to the disabled population should decline to a certain extent. This simulation decreased the three variables by 10%, 20% and 50%, respectively. The change was recorded in the four indicators of five states. It can be seen from the simulation result (Table 4) that the greater the stimulation degree, the more favorable the rapid decline of *RSR* scores, corresponding to a more effective policy. When the policy is weak (10% decrease), the decline of *RSR* value is very slow. Less effective social treatment may induce more opioid abuse. These simulation results point out the importance of raising the strategies and policies targeted at the disabled population. It may be inferred that the development of opioid abuse in the state would be significantly inhibited by adopting policies targeted at the disabled population. In the analysis conducted by Cordes, it was found that the mortality was positively correlated with the percentage of the disabled population [5]. In addition, the demographic pattern of deaths and the most rampant opioid drug type vary throughout time [30]. Thus, the model is needed to benefit policy making by evaluating the dynamic changes of socioeconomic factors and propose strategies for different specific populations.

## 4. Conclusions

In summary, this study presented the trends of the amount of drugs in five states of the United States. The heat map of the occurrence of opioid cases was plotted to illustrate the tendency of spreading. The overall growth rate was retarded. Some counties remained a slow growth rate. The advantage points distribution map characterized by the magnitude of the LOFC values was demonstrated. It turns out that the absolute advantage points were generally away from the state capitals. According to the scattering diagram, it may infer in that the counties, opioid abuse areas should exist surrounding the state capital. Additionally, we established the EWM-RSR model to analyze the severity of the opioid case abuse in these states. Combined with the AR model, the future development of opioid abuse was predicted. Furthermore, we used the LASSO regression for cross-validation to improve the model reliability with the optimal lambda value determined. Finally, the regression equation was added in the previous model to incorporate indicators related to demographic characteristics. It was found that the factors related to disabled people played a significant role in the level of opioid abuse. This combined model can be used to evaluate the policy effect on the opioid abuse control and provides instructions on curbing the trend of opioid abuse. Moreover, the proposed methods and models in this paper could be used for predicting the usage of other drugs given with previous data. The possibility of the area with a high risk of drug abuse could be estimated by identifying different indicators and calculating the sum of the rank defined by the model. Thus, the areas where drug abuse may occur could be determined. After determining the essential characteristic factors based on the socioeconomic data, some potentially modified policies could be assessed. Hence, it can help different agencies to perform effective measures to cope with drug-related challenges.

## Figures and Tables

**Table 1:** The 13 most serious opioid abuse counties.

State	County	$\rho_{i}$	$q_{i}$	$Q_{i}$	${\mathrm{LOFC}}_{k}\left(i\right)$	${\mathrm{RSR}}_{i}$
OH	39,061	0.37	3490.25	0.106	0.881	1.064
	39,113	0.32	885.25	0.102	0.742	0.964
	39,017	0.36	426	0.086	0.42	0.908
PA	42,003	0.48	1210.25	0.111	0.846	1.11
	42,081	0.35	110	0.175	0.672	0.995
KY	21,151	0.57	92.5	0.054	0.49	1.085
	21,067	0.38	93.75	0.099	0.66	0.989
	21,111	0.37	86.25	0.115	0.897	0.934
VA	51,187	0.52	36.5	0.098	0.418	1.072
	51,177	0.39	97.25	0.125	0.44	0.979
	51,047	0.33	47.75	0.19	0.442	0.927
WV	54,067	0.5	29.25	0.084	0.664	0.987
	54,033	0.47	31.25	0.236	0.546	0.89

**Table 2:** The indicators’ values of the counties with most serious opioid abuse.

State	County	${\mathrm{RSR}}_{2017}$	${\mathrm{RSR}}_{2018–2021}$	${\mathrm{Level}}_{2017}$	ΔLevel
OH	39,035	0.844	1.193	2	1
	39,085	0.872	1.000	2	1
PA	42,101	0.887	1.053	2	1
KY	21,059	0.592	1.007	3	2
	21,107	0.504	0.951	3	2
	21,227	0.474	1.107	4	3
VA	51,041	0.832	1.108	2	1
	51,121	0.671	1.226	3	2
	51,047	0.634	1.041	3	2
WV	54,107	0.833	1.028	2	1

**Table 3:** Main part of correlations and explanations.

$y_{i}$	$x_{i}$	Coefficient	Explanation
$\rho_{i}$	VC112	0.18404	The health of people who live out of a nursing home or institutions with medical instructions is not guaranteed. It is possible for them to access opioids through illegal channels. The disabled people may use opioids to alleviate physical or mental suffering which leads to opioid addiction.
	VC115	0.16771	
$q_{i}$	VC03	0.24625	If the proportion of drug users to the total population is fixed, a larger total number of households leads to more drug users.
$Q_{i}$	VC101	0.12905	Veterans should develop a certain degree of self-control through intensive training. They have a certain understanding of the harm of opioids. The increase in the proportion can lead to a less rapid growth of opioid usage.
	VC112	−0.25485	The healthcare of disabled civilians is not guaranteed for those who live out of a nursing home or institutions with medical instructions. The percentage of the population is relatively small among drug users due to finances and health conditions. They are less favored for opioids.
$LOFC_{k}\left(i\right)$	VC114	0.50514	People with disabilities are more likely to be exposed to opioids. Without appropriate medical guidance, the possibility of misuse or opioid abuse is high. The more people there are with disabilities in a county, the more likely it is to have opioid use. Therefore, a higher proportion of the disabled population leads to more opioid cases in the county, and a greater advantage ratio of the county relative to surrounding counties.
	VC122	0.23481	If a county has a small population flow range, the number of drug users may increase due to a gathering of drug users. As a result, the county may develop a greater advantage ratio of the county to surrounding counties.

**Table 4:** Results of policy change simulation.

Simulation Degree	Period	*RSR*	*RSR*
Base	N/A	0.60846	N/A
10%	1	0.60589	0.00257
	2	0.60461	0.00128
	3	0.60207	0.00254
	4	0.6008	0.00127
	5	0.59578	0.00502
20%	1	0.60461	0.00385
	2	0.6008	0.00381
	3	0.59205	0.00875
	4	0.58225	0.0098
	5	0.58104	0.00121
50%	1	0.59081	0.01765
	2	0.57383	0.01698
	3	0.56671	0.00712
	4	0.56553	0.00118
	5	0.56435	0.00118

## Data Availability

The data presented in this study are available as the Supplementary Materials.

## Supplementary Materials

The following are available online at https://www.mdpi.com/article/10.3390/healthcare9050585/s1. Table S1: Drug cases report released by National Forensic Laboratory Information System (NFLIS) in Ohio, Kentucky, West Virginia, Virginia, and Tennessee. Table S2: 2010 Population demographics in Ohio, Kentucky, West Virginia, Virginia, and Tennessee. Table S3: 2011 Population demographics in Ohio, Kentucky, West Virginia, Virginia, and Tennessee. Table S4: 2012 Population demographics in Ohio, Kentucky, West Virginia, Virginia, and Tennessee. Table S5: 2013 Population demographics in Ohio, Kentucky, West Virginia, Virginia, and Tennessee. Table S6: 2014 Population demographics in Ohio, Kentucky, West Virginia, Virginia, and Tennessee. Table S7: 2015 Population demographics in Ohio, Kentucky, West Virginia, Virginia, and Tennessee. Table S8: 2016 Population demographics in Ohio, Kentucky, West Virginia, Virginia, and Tennessee. Table S9: The description of the variables in the population demographic data. Table S10: All the correlation and explanations.
