# Supplementary material for: Evaluation of Policy Effectiveness by Mathematical Modeling for the Opioid Crisis with Spatial Study and Trend Analysis
Source: Healthcare (Basel). 2021 May 14;9(5):585. doi: 10.3390/healthcare9050585 (PMC8155830; doi:10.3390/healthcare9050585)
Supplement: Supplementary file 1 [file healthcare-09-00585-s001.zip › Supplementary table captions.pdf]

Excel Table S1 Drug cases report released by National Forensic Laboratory Information System (NFLIS) in Ohio, Kentucky, West Virginia, Virginia, and Tennessee.

Excel Table S2 2010 Population demographics in Ohio, Kentucky, West Virginia, Virginia, and Tennessee.

Excel Table S3 2011 Population demographics in Ohio, Kentucky, West Virginia, Virginia, and Tennessee.

Excel Table S4 2012 Population demographics in Ohio, Kentucky, West Virginia, Virginia, and Tennessee.

Excel Table S5 2013 Population demographics in Ohio, Kentucky, West Virginia, Virginia, and Tennessee.

Excel Table S6 2014 Population demographics in Ohio, Kentucky, West Virginia, Virginia, and Tennessee.

Excel Table S7 2015 Population demographics in Ohio, Kentucky, West Virginia, Virginia, and Tennessee.

Excel Table S8 2016 Population demographics in Ohio, Kentucky, West Virginia, Virginia, and Tennessee.

Excel Table S9 The description of the variables in the population demographic data.

Table S10 All the correlation and explanations.
