# Supplementary material for: Evaluation of Policy Effectiveness by Mathematical Modeling for the Opioid Crisis with Spatial Study and Trend Analysis
Source: Healthcare (Basel). 2021 May 14;9(5):585. doi: 10.3390/healthcare9050585 (PMC8155830; doi:10.3390/healthcare9050585)
Supplement: Supplementary file 1 [file healthcare-09-00585-s001.zip › Table S10.pdf]

Table S1 All the correlation and explanations

y_i	x_i	**Coefficient**	**Explanation**
ρ_i	VC17	0.03640	As the majority of the residents are under 18, and their knowledge of drugs are not enough, they are more likely addicted to drugs under the influence of other people. At the same time, opioids are more accessible to young people.
	VC48	0.06348	Divorced women have a lot of stress in life. In order to alleviate the depressed mood, some divorced women began to use opioids to seek spiritual relaxation and satisfaction.
	VC63	0.02294	Without children, the elderly with grandchildren have fewer restrictions on their children, and their drug education for children is relatively not enough, which increases the likelihood of children being exposed to opioids.
	VC112	0.18404	Non-institutionalized civilian life is not guaranteed. Coupled with disability, it is possible to gain access to opioids through illegal channels and even abuse opioids to alleviate physical or mental suffering and lead to opioid addiction.
	VC115	0.16771	The same reason as above
	VC135	-0.04632	People born in Puerto Rico, U.S. Island areas, or born abroad to American are more likely to smoke traditional drugs, and fewer people use opioids.
	VC162	0.05931	There may be more channels of exposure to drugs, including opioids, and becoming drug users.
	VC194	0.03767	Germany and Ireland are countries where drugs are rampant. Americans with German or Irish descent are likely to be affected by their family and use drugs, including opioids.
	VC197	0.22746	The same reason as above
q_i	VC03	0.24625	Under the situation that the proportion of drug users to the total population is fixed, the larger the total number of households, the more drug users.
Q_i	VC10	0.01721	Women’s income levels are relatively low, so opium drugs, which are relatively low-priced drugs, are favored, leading to a faster change in

			the proportion of opioids.
	VC11	0.05519	Women's income levels are relatively low, so opium drugs, which are relatively low-priced drugs, are favored, leading to a faster change in the proportion of opioids.
	VC18	0.03360	Female residents who live with their children alone have a relatively low income level, and higher spendings, thus cheaper drugs such as opioids are more favored, leading to a rapid change in the proportion of opioid drug uses.
	VC45	-0.03796	Married women account for a relatively small proportion of drug users, and a higher proportion of such people leads to a slower change in the proportion of opioids used.
	VC46	-0.00504	The economic situation of women separated from their husbands is generally not goof, with a smaller proportion of drug users and less favored with opioids.
	VC66	-0.00740	Without children, the elderly who live with grandchildren have fewer restrictions on their children, making some teenagers become drug addicts. However, because teenagers have no source of income, the trend of such drug users is slower.
	VC77	0.01399	The more children enrolled in kindergartens, the more families there are. In such a family, parents are more likely to reduce drug use or detoxification, resulting in a faster reduction in the proportion of opioids used.
	VC89	0.00964	People with higher education have a certain understanding of the dangers of opioids, and they have certain self-control ability. The increase in the proportion of such people will lead to a faster reduction in the proportion of opioids.
	VC101	0.12905	Veterans have a certain degree of self-control through strict training, and they also have a certain understanding of the harm of opioids. The proportion of such people has increased, making the use of opioids less rapid.
	VC112	− 0.25485	Non-institutionalized disabled civilians' lives are not guaranteed, its economic situation is relatively poor and their health conditions are not so good, thus their population is relatively

			small among drug users, and they are less favored for opioids.
	VC162	-0.01410	Drug users who are born in Asian Americans may be more inclined to use traditional drugs and have less favor for opioids. The increase in the proportion of such people may lead to a slower rate of change in the use of opioids.
	VC186	-0.22266	ANCESTRY - Total population - American
	VC196	0.08472	ANCESTRY - Total population - Hungarian
$LOFC_k(i)$	VC48	0.05785	MARITAL STATUS - Females 15 years and over - Divorced
	VC77	-0.02714	SCHOOL ENROLLMENT - Population 3 years and over enrolled in school - Nursery school, preschool
	VC88	-0.08638	EDUCATIONAL ATTAINMENT - Population 25 years and over - High school graduate (includes equivalency
	VC89	0.04705	EDUCATIONAL ATTAINMENT - Population 25 years and over - Some college, no degree
	VC114	0.50514	People with disabilities are more likely to be exposed to opioids. Without the correct guidance of doctors, the possibility of misuse or even abuse is high. Therefore, the more people with disabilities in a county, the more likely they are to have opioids. Therefore, the more disabled people there are in a county, the more likely there will be opioid cases, and the greater the advantage ratio of the county relative to surrounding counties.
	VC122	0.23481	If a county has a small population flow range, it is more likely to form a gathering place for drug users, which provides certain conditions for the increase of drug users in the county.Therefore, the county maybe have a greater advantage ratio of the county relative to surrounding counties.
	VC124	-0.11491	RESIDENCE 1 YEAR AGO - Population 1 year and over - Different house in the U.S. - Different county - Same state
	VC161	-0.02056	WORLD REGION OF BIRTH OF FOREIGN BORN - Foreign-born population, excluding population born at sea - Europe
	VC162	0.06079	WORLD REGION OF BIRTH OF FOREIGN BORN - Foreign-born population, excluding population born at sea - Asia

	VC194	-0.02320	ANCESTRY - Total population - German
